# Granger-Causality-Based Multi-Frequency Band EEG Graph Feature Extraction and Fusion for Emotion Recognition

**DOI:** 10.3390/brainsci12121649

**Published:** 2022-12-01

**Authors:** Jing Zhang, Xueying Zhang, Guijun Chen, Qing Zhao

**Affiliations:** College of Information and Computer, Taiyuan University of Technology, Taiyuan 030024, China

**Keywords:** emotion recognition, Granger causality, graph convolutional neural network, graph feature extraction, feature fusion

## Abstract

Graph convolutional neural networks (GCN) have attracted much attention in the task of electroencephalogram (EEG) emotion recognition. However, most features of current GCNs do not take full advantage of the causal connection between the EEG signals in different frequency bands during the process of constructing the adjacency matrix. Based on the causal connectivity between the EEG channels obtained by Granger causality (GC) analysis, this paper proposes a multi-frequency band EEG graph feature extraction and fusion method for EEG emotion recognition. First, the original GC matrices between the EEG signals at each frequency band are calculated via GC analysis, and then they are adaptively converted to asymmetric binary GC matrices through an optimal threshold. Then, a kind of novel GC-based GCN feature (GC-GCN) is constructed by using differential entropy features and the binary GC matrices as the node values and adjacency matrices, respectively. Finally, on the basis of the GC-GCN features, a new multi-frequency band feature fusion method (GC-F-GCN) is proposed, which integrates the graph information of the EEG signals at different frequency bands for the same node. The experimental results demonstrate that the proposed GC-F-GCN method achieves better recognition performance than the state-of-the-art GCN methods, for which average accuracies of 97.91%, 98.46%, and 98.15% were achieved for the arousal, valence, and arousal–valence classifications, respectively.

## 1. Introduction

Electroencephalogram (EEG) emotion recognition can help promote the interaction between humans and intelligent devices, holding an essential position in the field of human–computer interaction [1,2,3]. The emotion recognition system is usually composed of an emotion dataset, feature extraction and fusion, and an emotion recognition model. Among them, an effective emotion feature extraction and fusion method is one of the keys to improving the performance of emotion recognition. The spatial distribution of EEG electrode channels on the cerebral cortex is discrete and irregular, and also, there is connectivity among the EEG signals from different locations [4]. Therefore, this paper focuses on how to effectively utilize the information of the structure of the human brain and the causal relationships of EEG signals to carry out feature extraction and fusion.

Feature extraction plays an important role in EEG emotion recognition systems. From the perspective of traditional signal processing, the temporal features, frequency features, and time–frequency features of the EEG signals are usually extracted [5,6]. Since it is difficult to mine the deep emotional features contained in EEG signals, it is a challenge to further improve the performance of the emotion recognition task. With the development of deep learning, convolutional neural networks (CNN) have been widely applied in the field of emotion recognition by extracting the local features of the data through the convolution and pooling operations, learning deeper features with multiple convolutional layers [7,8,9]. In [7], a continuous convolutional neural network with four convolutional layers was designed to extract the deep features of EEG signals, which achieved 90.24% and 89.45% recognition accuracies for the arousal and valence classifications, respectively. In [8], CNN and long short-term memory modules were used to learn the spatial frequency and temporal features of EEG signals, and average binary classifications of 94.11% and 94.22% for arousal and valence was achieved, respectively. However, the limitation of CNNs is that the convolution operations are only effective for gridded data, such as pictures and videos. For example, References [7,8,9] mapped the EEG features into regular spaces before using the CNN models. In fact, the irregular spatial distribution of EEG signals means that they are non-gridded data. Therefore, CNNs cannot effectively deal with their connectivity, and they also have difficultly revealing the interaction among EEG channels. Fortunately, graph convolutional neural networks (GCNs) [10,11,12] combine the advantages of graph theory and CNNs, having great effectiveness in extracting the features of EEG signals with their irregular distribution.

GCNs are usually composed of nodes and adjacency matrices. For EEG signals, each EEG electrode channel is defined as a node, and the connections of any two nodes are defined as the adjacency matrix to construct the EEG graph features. In general, the nodes are fixed as the number of EEG electrodes is also fixed, and the adjacency matrix is not unique because of the different measures. The adjacency matrices of the current GCN method is mainly divided into two categories: One uses the spatial position relationship between the EEG channels [13,14,15], such as the typical Gaussian kernel function [13]. The other uses the connectivity of the brain [16,17,18], which can be further divided into functional connectivity and effective connectivity [19,20,21,22,23]. Functional connectivity is defined as the statistical interdependence among the EEG signals; most of the current research adopts functional connectivity matrices, such as the phase-locked value (PLV), Pearson correlation coefficient, and mutual information, as the adjacency matrices [16,17,19]. However, functional-connectivity-based adjacency matrices are symmetrical and ignore the directionality of the transmission of information in the brain. Different from functional connectivity, effective connectivity can further reveal the transmission of causal information in the brain system due to the addition of direction information. Granger causality (GC) is an effective connection measure [24,25,26]. In [26], the GC matrix was used as the adjacency matrix to construct the GCN feature, which can effectively improve the performance of the EEG recognition task. However, the current GCN features usually take the temporal or frequency features of each frequency band of the EEG signal as the nodes, but the functional or effective matrices of the full frequency band of the EEG signals ate taken as the adjacency matrices. As a result, the adjacency matrices cannot completely match the nodes, and the constructed graph features cannot accurately reflect the interaction between EEG signals in each frequency band. Therefore, this paper aims to construct a new GC-GCN feature by extracting the differential entropy (DE) features and GC matrix of each frequency band of the EEG signal, making full use of the causal relationship between the EEG signals to improve the performance of the emotion recognition task.

Research on cognitive neuroscience has shown that EEG signals in different frequency bands reflect various brain activities [27,28,29]. Generally, the EEG signal is decomposed into five bands: δ (0.5–4 Hz), θ (4–8 Hz), α (8–12 Hz), β (12–30 Hz), and γ (30–45 Hz). The existing feature fusion schemes are commonly used in series or in weighted superpositions of EEG features of different frequency bands to effectively improve the performance of the emotion recognition task. Methods such as phase-locked-value-based graph convolutional neural networks (PGCNNs) [19], dynamical graph convolutional neural networks (DGCNNs) [14], and causal graph convolutional neural networks (Causal-GCNs) [26] use a direct cascade of the different frequency band features as the nodes, which achieves a significant improvement over the single-frequency-band features. In [30], a frequency attention mechanism was applied to adaptively allocate weights to different frequency band features, with a 0.67% average improvement over the feature summation fusion scheme. However, the above feature fusion methods are carried out at the frequency band level, i.e., the features of all EEG channels in the same frequency band are assigned the same importance, ignoring the fact that the different channels of the EEG signals have different abilities for emotion recognition. Based on this, this paper proposes a new feature fusion scheme of the different frequency band features for the same node in GC-GCN graph features, which is an effective way to further improve the performance of emotion recognition.

As mentioned above, this paper mainly studies a GC-based multi-frequency band EEG graph feature extraction and fusion method. First, the original GC matrices between EEG signals at each frequency band are calculated through the GC method, and asymmetric binary GC matrices are obtained with an optimal threshold to adaptively select the larger causal value as connections. After this, the DE features and asymmetric binary GC matrices are used as the node values and the adjacency matrices to construct the GC-GCN graph features, respectively. Then, a new multi-frequency band feature fusion method (GC-F-GCN) is proposed by weighted integrating the GC-GCN features of EEG signals at each frequency band for the same node, which can effectively explore the frequency information that more significantly reflects the changes in emotional state. Finally, the recognition results of GC-F-GCN fusion features are obtained by using the Softmax classifier. Experimental results on the database for emotion analysis using physiological signals (DEAP) show that the proposed GC-F-GCN method can achieve better recognition performance.

The remaining parts are organized as follows. Section 2 reviews the GC theory and GCN network. Section 3 describes the proposed GC-F-GCN feature fusion method, including the construction of the GC-GCN graph feature and the multi-frequency band feature fusion scheme. Section 4 presents the experimental results and discussions. Finally, some conclusions are presented in Section 5.

## 2. Related Works

### 2.1. Overview of the GC Method

Granger causality is used to determine if causality exists between time series data, that is, whether a time series can be used to predict another. The GC-based EEG brain network has been widely used in the field of emotion recognition recently. Based on the definition of the GC method in [24], the vector autoregressive (VAR) model is applied to predict current value by using past time series values. Let X(t) and Y(t) be any two EEG series, X(t−i) and Y(t−i) represent the *i*-th time-lagged values of *X* and *Y*, respectively. Then, the univariate and the bivariate VAR models between *X* and *Y* can be expressed as:

(1) $$\left[\begin{array}{c} X(t)\\ Y(t) \end{array}\right]=\sum_{i=1}^{L}\left[\begin{array}{c} a_{1i}\\ b_{1i} \end{array}\right]\left[\begin{array}{c} X(t-i)\\ Y(t-i) \end{array}\right]+\left[\begin{array}{c} \varepsilon_{X}\left(t\right)\\ \varepsilon_{Y}\left(t\right) \end{array}\right]$$

(2) $$\left[\begin{array}{c} X(t)\\ Y(t) \end{array}\right]=\sum_{i=1}^{L}\left[\begin{array}{cc} a_{2i} & b_{2i}\\ c_{2i} & d_{2i} \end{array}\right]\left[\begin{array}{c} X(t-i)\\ Y(t-i) \end{array}\right]+\left[\begin{array}{c} \eta_{XY}\left(t\right)\\ \eta_{YX}\left(t\right) \end{array}\right]$$

where $a_{1i}$, $b_{1i}$, $a_{2i}$, $b_{2i}$, $c_{2i}$, and $d_{2i}\left(i=1,2,\ldots ,L\right)$ are the constant coefficients, and *L* is the lagged coefficient of the model. $\varepsilon_{X}\left(t\right)$ and $\varepsilon_{Y}\left(t\right)$ represent the prediction errors of the univariate VAR model through *X* and *Y*, respectively. $\eta_{YX}\left(t\right)$ and $\eta_{XY}\left(t\right)$ represent the prediction errors of the bivariate VAR model through *X* and *Y*, respectively.

By comparing the variance of the errors of the univariate and the bivariate VAR models, we can determine whether there is a GC relationship between *X* and *Y*. Therefore, the GC value is defined as the logarithm of the ratios of two variances of the errors:

(3) $$F_{X\to Y}=\ln\left(\frac{\sigma_{\varepsilon_{Y}}}{\sigma_{\eta_{XY}}}\right)$$

(4) $$F_{Y\to X}=\ln\left(\frac{\sigma_{\varepsilon_{X}}}{\sigma_{\eta_{YX}}}\right)$$

where $\sigma_{\varepsilon_{X}}$, $\sigma_{\varepsilon_{Y}}$, $\sigma_{\eta_{XY}}$, and $\sigma_{\eta_{YX}}$ represent the variances of the error.

As described in Equations (3) and (4), when $F_{X\to Y}>0$, means that *X* is the “Granger cause” to *Y*. Of course, when $F_{Y\to X}>0$, we say that *Y* is the “Granger cause” to *X*.

### 2.2. Overviews of Graph Convolutional Neural Networks (GCN)

Inspired by the convolution operations of CNNs for regular images, researchers have proposed graph convolution operations for graph-structured data in recent years. The existing GCN schemes are categorized into spectral graph convolution and spatial graph convolution by the types of graph filtering [31,32]. The spectral graph convolution first defines the graph’s Fourier transform. Then, it converts the graph data from the spatial domain to the frequency domain according to spectral graph theory and convolution theory with a solid theoretical foundation. The spatial graph convolution is defined as the process of directly iteratively aggregating a subset of neighbor nodes, which is more suitable for large-scale graph data. In this paper, the more common spectral-based GCN is applied.

Learning from the traditional Fourier-transform and convolution operations, GCNs define the graph convolution by defining the Laplacian matrix *L* on the graph. For a connected graph with *n* nodes G={V,E,A}, where *V*, *E*, and *A* denote the sets of nodes, edges, and the adjacency matrix, respectively. $A_{ij}\left(i,j=1,2,\ldots ,n\right)$ denotes the connection weights between node *i* and node *j*. The Laplacian matrix *L* is defined as

(5) L=D−A

where *D* is the degree matrix of *A*. The spectral decomposition of the Laplace matrix *L* is:

(6) $$L=U\Lambda U^{T}$$

where $\Lambda =\mathrm{diag}([\lambda_{1},\lambda_{2},\ldots \lambda_{n}])$ is a diagonal matrix, $U=[u_{1},u_{2},and\ldots u_{n}]$ is an orthogonal matrix that consists of the eigenvectors of *L*.

Let $g_{\theta }$ denote the graph filter and $\tilde{X}$ denote the output of graph convolution operation. The graph filter between *X* and $g_{\theta }$ can be calculated as follows:

(7) $$\tilde{X}=X*g_{\theta }\left(A\right)=Ug_{\theta }\left(\Lambda \right)U^{T}X$$

Due to the high computational complexity of $g_{\theta }(\Lambda )$, the Chebyshev polynomials are usually used in GCNs for the computation of the $g_{\theta }(\Lambda )$. In this case, the $g_{\theta }(\Lambda )$ can be expressed as:

(8) $$g_{\theta }(\Lambda )=\sum_{k=0}^{K}\theta_{k}T_{k}\left(\tilde{\Lambda }\right)$$

where $\theta_{k}$ are the coefficients of Chebyshev polynomials, $\tilde{\Lambda }=2\Lambda_{n}/\lambda_{\max}-I_{n}$ is a diagonal matrix, $T_{k}$ is a K-order Chebyshev polynomial. The filter process can be rewritten as:

(9) $$y=U\sum_{k=0}^{K}\theta_{k}g_{k}\left(\tilde{\Lambda }\right)U^{T}x=\sum_{k=0}^{K}\theta_{k}g_{k}\left(\tilde{L}\right)x$$

where $\tilde{L}=2L/\lambda_{\max}-I_{n}$.

In general, the dimensional transformation matrix *W* is applied to the filtered signal. Therefore, a complete graph convolution process can be expressed as:

(10) $$G\left(X,A\right)=X*g_{\theta }\left(A\right)W=\sum_{k=0}^{K}\theta_{k}T_{k}\left(\tilde{L}\right)XW$$

## 3. The Proposed GC-F-GCN Scheme for EEG Emotion Recognition

**Figure 1:**
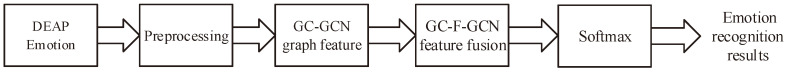
The framework of the proposed GC-F-GCN graph fusion feature method.

Figure 1 shows the framework of the proposed GC-F-GCN graph feature fusion method. The preprocessing module is first performed to the original EEG signal in the DEAP emotion dataset, such as downsampling, 4–45 Hz filtering, and re-referencing. The θ (4–8 Hz), α (8–12 Hz), β (12–30 Hz), and γ (30–45 Hz) frequency bands of EEG signals will be obtained through short-time Fourier transform (STFT). The GC-GCN graph feature module extracts the DE features and GC matrices of EEG signals at each frequency band as the nodes and adjacency matrices to construct the GC-GCN graph features. After that, the GC-F-GCN graph feature fusion module designs a new multi-frequency feature fusion scheme to integrate the GC-GCN features at different frequency bands for the same node. Finally, the Softmax layer module obtains the emotion recognition results.

### 3.1. Preprocessing of EEG Signals

The DEAP dataset [33] is an effective dataset that collects the EEG and peripheral physiological signals from 32 healthy participants. Before the experiment, all participants were informed in detail about the whole experiment. During the experiment, all the subjects watched 40 excerpts of one-minute duration music videos displaying different emotional states. The participants were asked to evaluate their levels of arousal, valence, linking, and dominance with self-assessment manikin (SAM). Finally, the EEG signals were recorded from 32 channels at a sampling rate of 512 Hz.

This paper only uses the EEG signals in the DEAP dataset for emotion recognition research. According to the 1 9 selfassessment scores of participants, we select the median score 5 as the threshold, with higher than 5 representing high class and less than or equal to 5 representing low class. The arousal space is divided into two parts: high arousal (HA) and low arousal (LA). The valence space is divided into two parts: high valence (HV) and low valence (LV). The valence-arousal (VA) space is divided into four parts, i.e., low arousal-low valence (LALV), high arousal-low valence (HALV), low arousal-high valence (LAHV), and high arousal-high valence (HAHV).

For the original EEG signal of each subject, the following processing was first performed: down-sampling to 128 Hz, removal of the electrooculogram (EOG) artifacts, bandpass filtering between 4 and 45 Hz, and removal of 3 s baseline. Then, each 60 s EEG signal was divided into $\left(\left(60-T_{w}\right)/T_{o}+1\right)$ segments with a window length of $T_{w}$ and an overlap time of $T_{o}$. Our previous work [34] has shown that the best recognition performance can be achieved when the $T_{w}$ and $T_{o}$ are 3 s and 1.5 s, respectively. EEG signals of four frequency bands were extracted using STFT. Finally, the DE features and the GC matrices were calculated for the EEG signals at each frequency band, where the GC matrix of EEG signals can be obtained from Equations (1)–(5), and the DE feature can be calculated as follows [35]:

(11) $$h\left(x\right)=\int_{-\infty }^{\infty }f(x)\log(f(x))dx$$

where *x* represents an EEG signal channel and f(x) is the probability density function of *x*. For an EEG signal that approximately obeys the Gaussian distribution, i.e., $x∼N(\mu ,\sigma_{i}^{2})$, the DE feature can be written as:

(12) $$h\left(x\right)=\int_{-\infty }^{\infty }\frac{1}{\sqrt{2\pi \sigma_{i}^{2}}}e^{\frac{{\left(x-\mu \right)}^{2}}{2\sigma_{i}^{2}}}\log\left(\frac{1}{\sqrt{2\pi \sigma_{i}^{2}}}e^{\frac{{\left(x-\mu \right)}^{2}}{2\sigma_{i}^{2}}}\right)dx=\frac{1}{2}\log\left(2\pi e\sigma_{i}^{2}\right)$$

### 3.2. The Construction of GC-GCN Graph Feature

**Figure 2:**
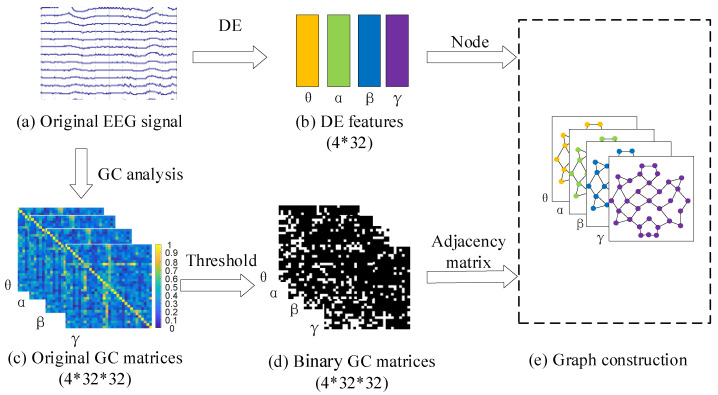
The construction process of the GC-GCN graph feature. (**a**) The original EEG signal. (**b**) The DE features. (**c**) The original GC matrices of the θ, α, β, and γ frequency EEG signal. (**d**) The binary GC matrices with the given threshold selection. (**e**) The final GC-GCN graph features.

As mentioned previously, the GCN features are composed of node values and adjacency matrices. In this paper, the DE features at each frequency band were used as nodes and the GC values between any two EEG signals were used as adjacency matrices to construct the GC-GCN graph features, which can effectively represent the spatial causal relationship between EEG electrode channels. As shown in Figure 2, the GC-GCN graph feature construction process in this paper is mainly divided into the following steps:

(1) Feature extraction. Figure 2a shows the original EEG signals with 32 channels. After the preprocessing described in Section 3.1, the DE features and the original GC matrices of EEG signals at θ, α, β, and γ frequency bands can be obtained, as shown in Figure 2b,c.

(2) Calculation of the GC-GCN adjacency matrix. Let $E_{i}$ and $E_{j}$(i,j=1,2,…,32, i≠j) denote any two EEG signals, and the GC values between them can be calculated from Equations (1)–(5). Therefore, the GC-GCN adjacency matrix $A_{GC}$ can be expressed as follows:

(13) $$A_{GC}=\left\{\begin{array}{cc} F_{ij}=\ln\left(\frac{\sigma_{\varepsilon_{j}}}{\sigma_{\eta_{ij}}}\right) & i>j\\ F_{ji}=\ln\left(\frac{\sigma_{\varepsilon_{i}}}{\sigma_{\eta_{ji}}}\right) & i<j\\ 1 & i=j \end{array}\right.$$

Figure 2c shows the original GC matrices $A_{GC}$, where the GC values of the EEG signals range from [0, 1]. The higher the GC value, the stronger the causality between the corresponding EEG signals. On the contrary, smaller GC values indicate weaker causal relationships or connections caused by noise [34]. Figure 2c shows that the number of causal values in the original GC matrix of EEG signals at each frequency band is 32×32. In this paper, we first sort these GC values from largest to smallest and then employ a threshold k(0<k<1) to obtain the set of the selected GC values, where the largest 32×32×k causal values are retained. In this way, the original GC matrix is converted into a binary GC matrix $A_{k}$, as described in Figure 2d and Equation (14).

(14) $$A_{k}=\left\{\begin{array}{cc} 1 & A_{GC}\left(i,j\right)\in X_{k}\\ 0 & A_{GC}\left(i,j\right)\notin X_{k} \end{array}\right.$$

(3) Construction of GC-GCN graph features. The DE features and the binary GC matrices $A_{k}$ of the EEG signals at each frequency band are used to construct the GC-GCN graph feature, as shown in Figure 2e.

### 3.3. Multi-Frequency Band Graph Feature Fusion Method

**Figure 3:**
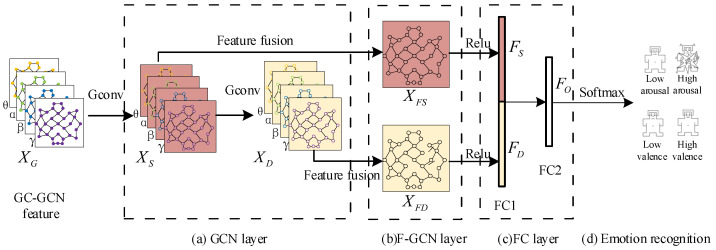
The framework of the proposed GC-F-GCN feature fusion method.

Based on the structural characteristics of GC-GCN graph features, we propose a new GC-F-GCN graph feature fusion method, which can adaptively integrate the GC-GCN graph features of the EEG signals at each frequency band for the same EEG node to better utilize the frequency information significantly associated with the different emotional states. The framework of the GC-F-GCN method is shown in Figure 3, which includes two graph convolution layers (GCN), a multi-frequency band feature fusion layer (F-GCN), two fully connected layers (FC), and a Softmax layer.

(a) GCN layer. By substituting the adjacency matrix $A_{k}$ in Equation (14) into Equation (10), we can obtain a complete graph convolution operation process described as Gconv in Figure 3. For the GC-GCN graph feature constructed in Figure 2, we first employ the Gconv operation on $X_{G}$ to extract the shallow graph features $X_{S}$, and then obtain the deep graph feature $X_{D}$ by the secondary Gconv operation. the $X_{S}$ and $X_{D}$ can be expressed as follows:

(15) $$X_{S}=G\left(X_{G},A_{k}\right)$$

(16) $$X_{D}=G\left(X_{S},A_{k}\right)$$

**Figure 4:**
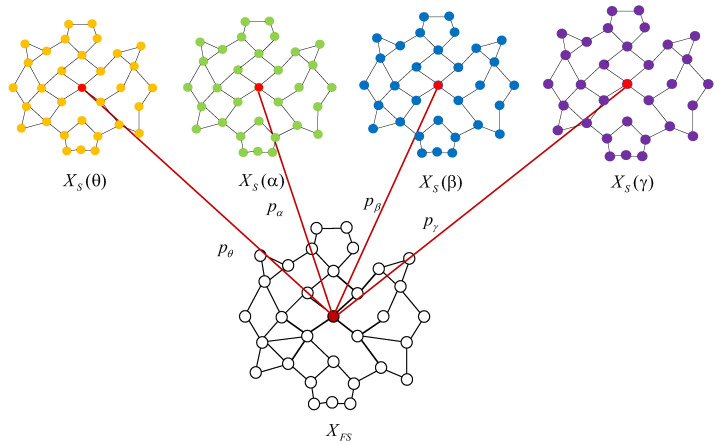
The fusion processes of the proposed F-GCN method for $X_{S}$.

(b) F-GCN layer. Considering the different emotion recognition abilities of the EEG features at the different frequency bands, a new multi-frequency band GC-GCN feature fusion method is designed for $X_{S}$ and $X_{D}$, which integrates GC-GCN features of the EEG signals at the θ, α, β, and γ frequency bands for the same EEG electrode channel. Since the fusion processes of $X_{S}$ and $X_{D}$ are similar, we will take $X_{S}$ as an example to describe the detailed fusion process, as shown in Figure 4.

As shown in Figure 4, let $X_{S}\left(\theta \right),X_{S}\left(\alpha \right),X_{S}\left(\beta \right),\;andX_{S}\left(\gamma \right)$ denote the shallow features of θ, α, β, and γ frequency EEG signals. $p_{\theta },p_{\alpha },p_{\beta },andp_{\gamma }$ denote the weight coefficients of θ, α, β, and γ frequency band GC-GCN features in the feature fusion process, respectively. $X_{FS}$ is the shallow fusion feature. Therefore, the fusion process of the F-GCN method can be expressed as Equation (17):

(17) $$X_{FS}=p_{\theta }X_{S}\left(\theta \right)+p_{\alpha }X_{S}\left(\alpha \right)+p_{\beta }X_{S}\left(\beta \right)+p_{\gamma }X_{S}\left(\gamma \right)$$

where $p_{\theta }+p_{\alpha }+p_{\beta }+p_{\gamma }=1$.

Similarly, the deep fusion features $X_{FD}$ can be calculated using Equation (17). To obtain the optimal weight coefficients $p_{\theta },p_{\alpha },p_{\beta },p_{\gamma }$, we employ the cross-entropy loss function to iteratively update the values of $p_{\theta },p_{\alpha },p_{\beta },p_{\gamma }$ in backpropagation, which will ensure the features with better recognition performance have a higher importance in the fusion features. The cross-entropy loss function can be expressed as:

(18) $$Loss=-\sum_{i=1}^{C}y_{i}\log{\hat{y}}_{i}$$

where *C* denotes the number of the emotional state, $y_{i}$ and ${\hat{y}}_{i}$ represent the actual emotional labels and the predicted emotional labels, respectively.

(c) FC layers. $X_{FS}$ and $X_{FD}$ are sequentially passed through the Relu activation function and FC1 layer, resulting in the FC shallow features $F_{S}$ and FC deep features $F_{D}$, respectively, as shown in Equations (19) and (20):

(19) $$F_{S}=f_{1}\left(Relu\left(X_{FS}\right)\right)$$

(20) $$F_{D}=f_{1}\left(Relu\left(X_{FD}\right)\right)$$

where $f_{1}$ represents the mapping process of FC1 layer, and the Relu activation function can be expressed as Relu(x)=max(x,0).

To ensure $F_{S}$ and $F_{D}$ have the same importance in the final emotion recognition process, we stipulate that $F_{S}$ and $F_{D}$ have the same dimensionality. The FC1 layer features $F_{S}$ and $F_{D}$ are then directly cascaded and integrated by the FC2 layer to obtain the final fusion feature $X_{o}$. This process can be expressed as:

(21) $$X_{o}=f_{2}\left(concat\left(F_{S},F_{D}\right)\right)$$

where $f_{2}$ represents the mapping process of the FC1 layer, concat represents the cascade features directly.

(d) Softmax layer. The final fusion feature $X_{o}$ is input into the Softmax layer to obtain the final emotion recognition results.

## 4. Experimental Results and Discussion

To evaluate the performance of the proposed scheme, we conducted three groups of experiments. In the first group, we discussed the influence of the threshold *k* in the GC-GCN graph feature and compared the emotion recognition performance of the GCN graph features with different adjacency matrices to verify the effectiveness of the GC-GCN graph feature. Next, we tested the performance of the proposed GC-F-GCN method through ablation experiments. Finally, we compared the emotion performances of the GC-F-GCN method with other state-of-art GCN methods. In addition, all the experiments were carried out on the computer with Nvidia GeForce RTX 1080 and Pytorch framework, and all the experimental results were averaged by 5-fold cross validation.

### 4.1. The Emotion Recognition Performance of the GC-GCN Graph Feature

As mentioned in Section 3.2, the GC matrices at different thresholds *k* correspond to different EEG topological connection structures. In this section, we mainly focus on the effect of the threshold *k* on the emotion recognition performance, where the threshold value *k* is chosen from 10% to 90%. Table 1 shows the emotion recognition performance of GC-GCN graph features with different thresholds *k*. It can be seen that the recognition performance of both binary and quadruple classifications is more sensitive to the threshold *k*. When the threshold values *k* are 60%, 30%, and 70%, the arousal, valence, and arousal–valence classifications show the best recognition performance, and the corresponding optimal average recognition accuracy values are 91.82%, 92.01%, and 87.38%, respectively. Therefore, only the optimal threshold *k* is used to calculate the GC adjacency matrix in the following section.

In addition, this section also compares three commonly used adjacency matrices for constructing GCN features: the random matrix, the shortest distance matrix, and the PLV-based matrix [19]. For fairness, all the recognition models adopted the same parameters and network structure. Table 2 shows the emotion recognition performance of the GCN graph features with different adjacency matrices.

(1) For the single-frequency-band EEG signals, the emotion recognition accuracy of the proposed GC-GCN graph feature is 4–14% higher than that of the random-based, the shortest distance-based, and PLV-based GCN graph features in the arousal, valence, and arousal–valence classifications.

(2) For the full-frequency-band EEG signals, the emotion recognition accuracies of proposed GC-GCN graph features are 91.82%, 92.01% and 87.38% for arousal, valence, and arousal–valence classifications. Compared to the GCN graph features constructed by the random matrix, shortest distance matrix, and PLV-based matrix, the GC-GCN features can achieve average improvements of 4.37%, 4.95%, and 0.93% for binary classifications of arousal, 4.70%, 4.43%, and 1.66% for binary classifications in valence, and 3.50%, 3.52%, and 1.74% for four classifications in arousal–valence, respectively.

(3) The paired samples t-test is utilized to verify whether there were significant differences in classification performance between GC-GCN graph features and other methods. The results also show that the GC-GCN graph features can obtain significantly higher accuracy (PLV: *p* = 0.014 < 0.05, others *p* < 0.01) in the arousal classification, (*p* < 0.01) in the valence classification, and (PLV: *p* = 0.015 < 0.05, others *p* < 0.01) in the arousal–valence classification, respectively.

### 4.2. The Performance of the GC-F-GCN Feature Fusion Method

In this section, we perform ablation experiments on the GC-F-GCN feature fusion method to analyze the contribution of each module, including the following three ablation experiments:

(1) The shallow graph feature (SGC-GCN) and the shallow fusion feature (SGC-F-GCN);

(2) The deep graph feature (DGC-GCN) and the deep fusion feature (DGC-F-GCN);

(3) The shallow and deep cascade graph feature (SDGC-GCN) and the shallow and deep fusion feature (SDGC-F-GCN).

Table 3 shows the emotion recognition performance of the ablation experiments performed using the GC-F-GCN method, from which the following conclusions can be obtained:

(1) The emotion recognition performance of the fusion graph features is significantly improved than the original graph features. For SGC-GCN shallow features, the average recognition performance with SGC-F-GCN shallow fusion features is 4.97%, 6.40%, and 9.60% higher than that of arousal, valence, and arousal–valence classifications, respectively. For the DGC-GCN deep features, the average recognition performance with DGC-F-GCN deep fusion features is 5.59%, 6.22%, and 10.40% higher than that of arousal, valence, and arousal–valence classifications, respectively. Otherwise, the average recognition performance of GC-F-GCN fusion features is 4.33%, 5.21%, and 5.77% higher than that of SDGC-GCN features in arousal, valence, and arousal–valence classifications, respectively.

(2) The GC-F-GCN graph features achieve a significant improvement over the single fusion graph features. Specifically, compared with the SGC-F-GCN and DGC-F-GCN features, GC-F-GCN features improved by 0.79% and 0.50% in arousal classification, 1.35% and 0.23% in valence classification, and 1.02% and 0.37% in arousal–valence classification, respectively.

(3) The paired samples t-test is utilized to verify whether there were significant differences in classification performance between these GC-GCN graph features. The results show that the SDGC-GCN graph features can obtain significantly higher accuracy (SGC-GCN: *p* = 0.044 < 0.05, DGC-F-GCN: 0.015 < 0.05, and others *p* < 0.01) in the arousal classification, (DGC-F-GCN: 0.034 < 0.05, and others *p* < 0.01) in the valence classification, and (SGC-GCN: 0.046 < 0.05, SDGC-GCN: 0.045 < 0.05, DGC-F-GCN: 0.041 < 0.05, and others *p* < 0.01) in the arousal–valence classification, respectively.

### 4.3. Performance Comparisons for Latest GCN Graph Feature

In this section, we compare the proposed scheme with other state-of-the-art graph features in the literature, and the comparison results are shown in Table 4, including the support vector machine (SVM), artificial neural network (ANN), DGCNN [14], PGCNN [19], and Causal-GCN [26]. All the schemes adopt the same EEG signal division and 5-fold cross validation. The parameter numbers of the network and the average training time of the corresponding recognition model are presented in Table 5.

We make the following statements based on the results:

(1) Compared with the DGCNN, PGCNN, and causal-GCN graph features, the GC-GCN graph features can achieve an average improvement of 1% to 5% in arousal, valence, and arousal–valence classifications, the average parameter numbers of the corresponding recognition model are almost the same, and the average training time increased by about 60%, while the

(2) Compared with the DGCNN, PGCNN, and causal-GCN graph features, GC-F-GCN fusion features are increased by 9.35%, 9.42%, and 9.43% in arousal classification, 10.10%, 10.20%, and 10% in valence classifications, and 15.75%, 15.50% and 15.80% in arousal–valence classifications, respectively.

(3) The GC-F-GCN fusion features are 6–10% higher than the GC-GCN graph features. The average parameter numbers and the training time of the recognition model increased by about 492% and 335%, respectively. Compared with the SVM and ANN classifiers, the GC-F-GCN method is increased by 13.71% and 5.04% in arousal classification, 15.03% and 6.18% in valence classifications, and 10.47% and 10.62% in arousal–valence classifications, respectively. This further demonstrates that the proposed model has a better recognition performance.

(4) The paired samples t-test is utilized to verify whether there were significant differences in classification performance between GC-GCN graph features and other methods. The results show that the GC-GCN graph features can obtain significantly higher accuracy (*p* < 0.01) both in the arousal, valence, and arousal–valence classifications, respectively.

## 5. Discussions

For the adjacency matrix of the GCN graph features of EEG signals, compared with the spatial position relationship between the EEG channels [13,14,15] and functional-connectivity-based adjacency matrices [16,17,19], GC-based adjacency matrices provide potential possibility (i.e., offering more direction information) to improve the recognition accuracy for the emotion-based system. In our work, the GC-based GCN graph feature shows the superiority to the other matrices, which is consistent with the reference [26].

In this paper, we discuss the emotion recognition performance of three commonly used adjacency matrices for constructing GCN features with the proposed GC-GCN graph features in Table 2. It can be seen that the proposed GC-GCN graph features achieve the best recognition performance in both the single frequency band and full frequency band. This is because the adjacency matrix of GC-GCN features not only utilizes the directionality of the causal information flow between EEG channels but also constructs GC-GCN graph features for each frequency EEG signal, making the nodes match the adjacency matrix, which is more suitable for the cognitive mechanism of the brain. We could also observe that the emotion recognition performance of the full frequency band EEG signal is significantly better than that of the single frequency band EEG signals. Previous studies also have shown that the full frequency band is more comprehensive and effective than a single frequency band [27,30]. Therefore, the complementary between the GCN graph feature of the EEG signals at the different frequency bands can be effectively used to improve emotion recognition performance effectively.

To analyze the contribution of each module of the GC-F-GCN feature fusion method, the ablation experiments are presented in Table 3. Compared with the original graph features, the GC-F-GCN scheme has significant improvements in arousal, valence, and arousal–valence classifications, respectively. For the original SGC-GCN and DGC-GCN graph features, the corresponding SGC-F-GCN and DGC-F-GCN graph fusion features can be obtained by using the frequency fusion module only once. The experimental results in Table 3 indicate that using the frequency fusion module on the original GC-GCN features can effectively improve the EEG signal emotion recognition performance.

Finally, several state-of-the-art GCN features are used to verify the validity of the proposed GC-F-GCN graph features, such as PGCNN [14], DGCNN [19], and Causal-GCN [26]. The results in Table 4 show that the GC-F-GCN graph feature can effectively improve the performance of emotion recognition than the other GCN graph feature. This is because the DGCNN only considers the spatial location relationship of the EEG signals, the PLV-based adjacency matrix of the PGCNN feature is undirected, and both of them ignore the fact that the connections between EEG channels are directional. The causal-GCN feature only adopts the GC matrix of the full-frequency EEG signals to obtain the adjacency matrix, making the nodes in the constructed GCN features mismatch with the adjacency matrices. The GC-F-GCN method constructs GC-GCN features for each frequency band EEG signal and integrates the shallow and deep graph features, which makes the emotion-related information in the EEG signal can be better extracted. In addition, the increased parameters and training time of the GC-F-GCN method mainly come from additional F-GCN layers and FC layers, which are the essential costs of recognition performance improvement.

## 6. Conclusions

In this paper, a novel multi-frequency band graph feature extraction method and fusion method are proposed for EEG emotion recognition, which simultaneously considers the spatial, frequency, and causal information of emotional EEG signals. First, the GCN adjacency matrix was constructed by setting the threshold value, and the larger causal values in the GC matrix of the EEG signals at each frequency band were selected adaptively, which can preserve more significant causal connections between EEG channels and make the selected EEG connections more suitable for brain cognitive patterns. Then, the DE features and selected binary GC matrix of the EEG signals at each frequency band signal were applied as node values and adjacency matrices to construct the GC-GCN graph feature. Finally, based on the structural characteristics of GC-GCN graph features, a new multi-frequency band EEG feature fusion method was proposed to integrate the graph information of EEG signals at different frequency bands for the same node, which can further improve the performance of emotion recognition. Experimental results show that the emotion recognition accuracy of the proposed GC-F-GCN scheme is 97.91% and 98.46% for the arousal and valence binary classifications, while 98.15% for the arousal–valence classification, respectively. In future work, we want to try to combine the spatial attention mechanism with the GC-F-GCN method to explore the brain regions significantly associated with human emotions, which would further improve the ability of the EEG emotion recognition system.

## Figures and Tables

**Table 1:** Emotion recognition performance of GC-GCN graph features with different threshold *k*.

*k*	Arousal	Valence	Arousal–Valence
10%	91.35 ± 5.37	91.34 ± 5.45	86.90 ± 8.23
20%	91.56 ± 5.14	91.66 ± 5.25	87.21 ± 8.31
30%	91.22 ± 5.53	92.01 ± 5.23	86.71 ± 8.10
40%	91.15 ± 5.34	91.23 ± 5.52	86.93 ± 8.45
50%	91.55 ± 5.33	91.46 ± 5.57	87.31 ± 8.78
60%	91.82 ± 4.82	91.27 ± 5.88	86.80 ± 9.1
70%	91.73 ± 5.66	91.25 ± 4.79	87.38 ± 7.94
80%	91.48 ± 5.21	91.91 ± 4.88	87.30 ± 9.06
90%	91.38 ± 5.26	91.60 ± 5.09	86.87 ± 8.91

**Table 2:** The recognition performance of the GCN features with different adjacency matrices (%).

Emotion	Adjacency Matrix	θ	α	β	γ	θ+α+β+γ
Arousal	Random	71.89 ± 5.44	72.12 ± 5.80	71.22 ± 5.78	71.54 ± 4.87	87.45 ± 5.53
	Distance	71.42 ± 5.93	72.52 ± 6.24	75.32 ± 6.24	75.10 ± 5.74	87.23 ± 5.17
	PLV [19]	72.31 ± 5.24	71.78 ± 5.17	71.44 ± 4.80	71.95 ± 5.58	90.89 ± 4.68
	GC-GCN	72.58 ± 5.16	78.09 ± 5.94	78.68 ± 5.82	79.55 ± 5.56	91.82 ± 4.82
Valence	Random	69.92 ± 6.46	69.71 ± 6.65	69.42 ± 6.50	69.80 ± 5.46	87.31 ± 4.75
	Distance	70.61 ± 6.36	70.67 ± 6.26	70.33 ± 7.12	69.89 ± 5.75	87.58 ± 5.42
	PLV [19]	69.94 ± 6.22	69.34 ± 6.24	70.56 ± 6.33	70.50 ± 5.83	90.35 ± 5.02
	GC-GCN	78.06 ± 5.84	78.25 ± 5.01	76.85 ± 6.37	77.66 ± 5.45	92.01 ± 5.23
Arousal–Valence	Random	55.92 ± 9.24	54.35 ± 8.34	53.80+9.38	55.34+9.29	83.88 ± 9.00
	Distance	54.51 ± 9.57	54.90 ± 8.28	54.00 ± 8.40	55.27 ± 9.57	83.86 ± 8.66
	PLV [19]	53.76 ± 8.96	54.88 ± 9.51	70.36 ± 6.22	71.07 ± 70.1	85.64 ± 7.99
	GC-GCN	71.47 ± 4.93	72.07 ± 5.07	71.97 ± 4.36	72.09 ± 5.19	87.38 ± 7.94

**Table 3:** Emotion recognition performance of GC-GCN graph features with different threshold *k*.

Features	Arousal	Valence	Arousal–Valence
SGC-GCN	92.12 ± 3.06	90.71 ± 5.00	87.53 ± 2.91
DGC-GCN	91.82 ± 4.82	92.01 ± 5.23	87.38 ± 7.94
SDGC-GCN	93.58 ± 5.78	93.25 ± 4.22	92.38 ± 3.05
SGC-GCN	97.12 ± 2.53	97.11 ± 2.30	97.13 ± 2.66
DGC-GCN	97.41 ± 3.29	98.23 ± 1.38	97.78 ± 3.26
SDGC-F-GCN	97.91 ± 2.50	98.46 ± 1.16	98.15 ± 2.39

**Table 4:** The recognition performance with latest schemes (%).

Emotion	Method	θ	α	β	γ	θ+α+β+γ
Arousal	SVM	74.19 ± 8.55	76.05 ± 8.68	80.10 ± 8.47	83.74 ± 7.17	84.20 ± 9.39
	ANN	77.72 ± 10.82	89.60 ± 7.73	87.14 ± 8.38	86.67 ± 7.01	92.87 ± 5.45
	DGGCN [14]	72.63 ± 5.59	72.33 ± 5.77	72.23 ± 5.75	72.43 ± 5.85	88.56 ± 5.98
	PGCNN [19]	72.31 ± 5.24	71.78 ± 5.17	71.44 ± 4.80	71.95 ± 5.58	90.89 ± 4.68
	Causal-GCN [26]	72.24 ± 5.41	71.93 ± 5.19	72.15 ± 4.98	72.61 ± 5.20	88.48 ± 5.71
	GC-GCN	72.58 ± 5.16	78.09 ± 5.94	78.68 ± 5.82	79.55 ± 5.56	91.82 ± 4.82
	GC-F-GCN	84.14 ± 7.61	93.45 ± 5.42	92.62 ± 5.60	91.69 ± 4.72	97.91 ± 2.50
Valence	SVM	67.68 ± 9.42	72.79 ± 9.14	77.17 ± 11.80	78.76 ± 11.02	83.43 ± 11.50
	ANN	78.63 ± 10.13	89.65 ± 6.81	87.73 ± 6.07	87.90 ± 5.38	92.28 ± 6.12
	DGGCN [14]	72.64 ± 5.49	72.25 ± 5.94	71.79 ± 4.86	72.27 ± 5.79	88.36 ± 5.48
	PGCNN [19]	69.94 ± 6.22	69.34 ± 6.24	70.56 ± 6.33	70.50 ± 5.83	90.35 ± 5.02
	Causal-GCN [26]	71.92 ± 5.63	71.95 ± 5.36	71.69 ± 5.40	71.96 ± 5.62	88.46 ± 5.11
	GC-GCN	78.06 ± 5.84	78.25 ± 5.01	76.85 ± 6.37	77.66 ± 5.45	92.01 ± 5.23
	GC-F-GCN	84.02 ± 8.01	84.19 ± 7.86	83.98 ± 7.83	83.82 ± 7.76	98.46 ± 1.16
arousal–Valence	SVM	69.54 ± 14.10	78.61 ± 11.44	82.31 ± 9.67	86.95 ± 7.89	87.68 ± 6.98
	ANN	69.03 ± 13.05	68.64 ± 14.39	62.34 ± 16.20	60.78 ± 14.05	87.53 ± 12.25
	DGGCN [14]	72.65 ± 5.60	73.15 ± 5.86	72.65 ± 5.59	73.00 ± 5.29	82.40 ± 9.32
	PGCNN [19]	53.76 ± 8.96	54.88 ± 9.51	70.36 ± 6.22	71.07 ± 70.1	85.64 ± 7.99
	Causal-GCN [26]	73.10 ± 5.39	73.22 ± 5.37	73.14 ± 5.37	73.39 ± 6.29	82.35 ± 9.23
	GC-GCN	71.47 ± 4.93	72.07 ± 5.07	71.97 ± 4.36	72.09 ± 5.19	87.38 ± 7.94
	GC-F-GCN	77.28 ± 11.96	77.20 ± 12.68	77.07 ± 12.10	77.05 ± 12.32	98.15 ± 2.39

**Table 5:** The parameter numbers of network and average training time of different GCN scheme.

Model	Arousal		Valence		Arousal–Valence	
	Parameters	Times (s)	Parameters	Times (s)	Parameters	Times (s)
DGGCN [14]	4338	15	4338	15	4404	16
PGCNN [19]	4274	16	4274	16	4340	16
Causal-GCN [26]	4402	17	4402	17	4468	18
GC-GCN	4218	25	4218	26	4284	29
GC-F-GCN	4970	123	24,970	124	25,076	126

## Data Availability

The database used in this study is publicly available at websites: DEAP: http://www.eecs.qmul.ac.uk/mmv/datasets/deap/, accessed on 10 October 2022.

## Abbreviations

The following abbreviations are used in this manuscript:

EEG	Electroencephalogram
GC	Granger causality
GCN	Graph convolutional neural network
GC-GCN	GC-based GCN graph feature
GC-F-GCN	Multi-frequency band GC-GCN feature fusion method
PLV	Phase Locking Value
SGC-GCN	Shallow graph feature
DGC-GCN	Deep graph feature
SDGC-GCN	Shallow and deep cascade graph feature
SGC-F-GCN	Shallow fusion feature
DGC-F-GCN	Deep fusion feature
SDGC-F-GCN	Shallow and deep fusion feature
